# E-cigarette use among female Chinese Indonesian college students in China: A qualitative interpretive phenomenological analysis from an acculturation perspective

**DOI:** 10.18332/tid/219000

**Published:** 2026-05-14

**Authors:** Yu Chen, Yan Wang, Zhangyan Li, Xinjie Zhao, Xinyao Yu, Ying Yue, Kin-Sun Chan

**Affiliations:** 1 School of Art and Communication, Fujian Polytechnic Normal University, Fuzhou, China; 2 School of Media and International Culture, Zhejiang University, Hangzhou, China; 3 School of Journalism and Communication, Peking University, Beijing, China; 4 Faculty of Social Sciences, University of Macau, Macau, China; 5 China National Center for Food Safety Risk Assessment, Beijing, China

**Keywords:** e-cigarettes, acculturation, international students, qualitative research

## Abstract

**INTRODUCTION:**

International students navigate health behaviors across different cultural and regulatory environments, yet their e-cigarette use remains understudied. This qualitative study explores e-cigarette use among female Chinese Indonesian college students in China, examining how acculturation experiences and gendered social norms shape vaping behaviors.

**METHODS:**

This qualitative study was conducted at a university in Fujian Province, China, in October 2024, employing Interpretive Phenomenological Analysis (IPA). Three female Chinese Indonesian college students (aged 18–21 years) were purposively selected from a larger survey (n=45) based on Indonesian nationality with Chinese ancestry, female gender, current e-cigarette use, and willingness to participate. Semi-structured interviews were supplemented by questionnaire data assessing acculturation difficulties, psychological well-being (CES-D-10), and social support. Analysis followed Smith's IPA procedures with Berry's acculturation framework as an interpretive lens.

**RESULTS:**

Four main themes emerged: 1) Social initiation and peer-embedded use – initiated through peer influence in Indonesian high schools and maintained within co-national networks in China; 2) Acculturation stressors without stress-coping use – participants described e-cigarette use as unrelated to academic pressure, language barriers, and loneliness; 3) Navigating gendered social norms – China was perceived as more permissive, with use concealed from families; and 4) Habituated practice and flavor preferences – use was framed as habit, with fruit flavor preferences driving cross-border sourcing.

**CONCLUSIONS:**

E-cigarette use among these students appears to be shaped by peer socialization and habit formation rather than acculturative stress; future longitudinal, multi-site studies with larger samples are needed to examine these patterns among broader international student populations.

## INTRODUCTION

Electronic cigarettes (e-cigarettes) have emerged as a significant global public health concern, particularly among young people. The rapid proliferation of e-cigarettes, combined with flavored products appealing to youth, has prompted calls for stronger regulatory frameworks^1^. While e-cigarette use has been examined extensively among domestic populations in both Western and Asian contexts, international students – who navigate health behaviors across different cultural, regulatory, and social environments – remain understudied.

The global population of international students exceeded 6 million before the COVID-19 pandemic, representing a significant mobile population whose health behaviors warrant attention^2^. International students face unique challenges, including language barriers, academic adjustment, and social isolation, that may influence health behaviors^3^. Research has documented that international students experience elevated stress levels, sleep difficulties, and academic performance concerns during acculturation, with these stressors potentially affecting health-related behaviors^4^.

Research examining tobacco use among international students has begun to emerge. Kim et al.^5^ conducted a comprehensive review of tobacco use among Asian Americans and found that acculturative stress, social networks, and cultural norms significantly influenced smoking behaviors, with peer influence emerging as a particularly salient factor. Firth et al.^6^ documented increased e-cigarette use among US college students during study abroad periods, suggesting cross-cultural transitions may represent periods of behavioral vulnerability. However, research specifically examining e-cigarette use among international students in non-Western contexts remains limited. No prior study has examined e-cigarette use among female, heritage-linked international students who navigate gendered stigma originating from their home country alongside perceived host-country permissiveness and divergent e-cigarette product regulation – a population at the intersection of gender, migration, cultural heritage, and tobacco regulatory disparities.

The Berry^7^ acculturation model provides a foundational framework for understanding cultural adaptation processes and their health implications. The model recognizes that adaptation involves both cultural learning and stress responses, with documented associations between acculturation and tobacco use that vary by gender and ethnicity^8^. Recent theoretical developments have emphasized the multidimensional nature of acculturation, recognizing that individuals may selectively adopt aspects of host cultures while maintaining heritage cultural practices.

Research on Asian immigrants in Western contexts has documented an ‘acculturation paradox’ for women: higher acculturation is associated with increased smoking among women but decreased smoking among men^9,10^. This pattern has been attributed to the relaxation of traditional gender-based prohibitions against female smoking. Whether similar dynamics operate for international students – particularly those studying in ancestral homelands – remains unexplored.

This study focuses on Chinese Indonesians – Indonesian citizens of Chinese descent – who are studying in China. Chinese Indonesians constitute approximately 1.2% of Indonesia’s population and maintain varying degrees of cultural connection to Chinese heritage^11^.

Indonesia presents a critical context for tobacco research. Indonesia’s tobacco regulatory environment is characterized by the absence of FCTC ratification, limited restrictions on tobacco advertising and promotion, the lack of pictorial health warnings on e-cigarette products, and inconsistent enforcement of age-of-sale regulations for both conventional and electronic cigarettes^1^. Indonesia has among the highest male smoking rates globally (66.0%) alongside very low female smoking rates (2.1%)^1^. Female tobacco use is strongly stigmatized in Indonesian society, reinforced by intersecting cultural gender norms and religious considerations. While Indonesia has the world’s largest Muslim population and several prominent Islamic organizations (e.g. Muhammadiyah, Nahdlatul Ulama) have issued ‘fatwas’ discouraging or prohibiting smoking, these positions vary in scope and application^12,13^. More relevant to the acculturation framework, these religious and cultural norms contribute to a broader normative environment of gendered respectability expectations, family surveillance, and anticipated stigma that shape how female tobacco use is practiced and concealed. Chinese Indonesians, while predominantly non-Muslim, exist within this broader normative environment.

While the broader literature on acculturation and tobacco often addresses combustible cigarettes, this study focuses specifically on e-cigarette use, which differs from conventional smoking in device technology, flavor availability, sourcing channels, social perceptions, and regulatory treatment. Where references to ‘tobacco use’ appear, they refer to the broader behavioral category relevant to the cited literature; findings and interpretations pertain specifically to e-cigarette use unless otherwise stated.

China has ratified the FCTC and implemented various tobacco control measures, though enforcement has been variable^14^. In October 2022, China enacted regulations restricting e-cigarette flavors to tobacco-only, creating regulatory divergence from Indonesia’s largely unregulated e-cigarette market^15^. For Chinese Indonesian students studying in China, acculturation involves navigating heritage-linked norms (Indonesian gendered respectability expectations, family surveillance norms, religious-cultural prohibitions on female tobacco use) alongside host-country conditions (perceived permissiveness toward female smoking, different regulatory frameworks, co-national peer networks that may reproduce or transform home-country behavioral norms). The framework of Berry^7^directs attention to how these heritage and host orientations interact – through peer networks, regulatory environments, family communication patterns, and shifting identity negotiations – to shape the practice and meaning of e-cigarette use in the cross-cultural transition.

This study aims to examine e-cigarette use among female Chinese Indonesian college students in China. The research addresses the questions: 1) ‘How do participants describe and understand their e-cigarette use experiences?’; 2) ‘How do participants make sense of their e-cigarette use in the context of acculturation experiences, including academic adjustment, language barriers, social support, and psychological well-being?’; 3) ‘How do participants perceive and navigate gendered social norms regarding e-cigarette use across Indonesian and Chinese contexts?’; and 4) ‘What role do peer networks play in initiating and maintaining e-cigarette use?’.

## METHODS

### Study design, setting, and methodological orientation

This qualitative study was conducted at a university in Fujian Province, China, during October 2025. The university is located in a region with historical significance for overseas Chinese emigration to Southeast Asia and hosts a substantial population of Chinese Indonesian students. The timing was approximately three years after China implemented e-cigarette flavor restrictions (October 2022), providing relevant regulatory context.

The study employed Interpretive Phenomenological Analysis (IPA), an approach that examines how individuals make sense of significant life experiences^16^. IPA is grounded in phenomenology, hermeneutics, and idiography, and is appropriate for small, homogeneous samples where depth of analysis takes precedence over breadth^17^. The acculturation framework of Berry^7^ served as a sensitizing theoretical lens, guiding attention to cultural adaptation processes while maintaining openness to participants’ own meaning-making.

This study adhered to the Standards for Reporting Qualitative Research (SRQR) guidelines^18^. The complete SRQR checklist is provided in the Supplementary file.

### Ethical considerations

This study received ethical approval from the Research Ethics Committee of Fujian Polytechnic Normal University (Approval No. 2025-02). All participants provided written informed consent before participation, including consent to audio recording and the publication of anonymized data. Given the sensitive nature of tobacco use data, particularly for female participants from cultures where female smoking is stigmatized, additional confidentiality protections were implemented. Participants were assured their information would not be shared with university administrators or family members. All identifying information has been removed; participants are identified as Participant 1 (P1), Participant 2 (P2), and Participant 3 (P3) throughout.

### Research team and reflexivity

The primary researcher is a Chinese female academic with experience in health communication research. She conducted all interviews and analysis. The researcher has no personal history of tobacco or e-cigarette use and approached participants’ accounts with openness and curiosity rather than judgment. Before interviews, the researcher disclosed her academic role and research purposes to participants. No prior relationship existed between the researcher and participants. Throughout the analysis, the researcher maintained reflexive awareness of her position as a non-smoker and as someone from a different cultural background than the participants, using a reflexive journal to document analytical decisions and interpretive choices.

### Sampling strategy

Participants were recruited from a larger survey examining health and cultural adaptation among heritage students at the university (n=45; Chan 2025, unpublished institutional survey). Purposive sampling identified individuals meeting the inclusion criteria: 1) Indonesian nationality with Chinese ancestry; 2) female gender; 3) current e-cigarette use (defined as any e-cigarette use in the past 30 days); and 4) willingness to participate in in-depth interviews. Three participants met all criteria and consented to participate.

The sample size of three is consistent with IPA methodology recommendations. Smith et al.^16^ state that IPA studies typically include 3–6 participants, emphasizing depth over breadth. Malterud et al.^19^ information power concept supports smaller samples when specificity, clarity of the theoretical framework, dialogue quality, and analytical depth are strong – all characteristics present in this study, given the highly specific population and the substantial interview depth achieved.

### Data collection methods

Before interviews, participants completed a comprehensive questionnaire. Questionnaire data served exclusively as individual-level contextual profiles to enrich the interpretive analysis of each participant’s interview account; they are not used for statistical inference or generalization. The questionnaire assessed the following: demographics and cultural identity (age, year of study, generation of Chinese descent, ancestral province, religion, and home language, defined as the primary language used at home in Indonesia); acculturation difficulties (20 items covering climate, food, social relationships, language, academic adjustment, and cultural understanding, rated on a 5-point scale from 1=no difficulty to 5=very difficult); social support (13-item scale adapted from the Multidimensional Scale of Perceived Social Support, rated 1=strongly disagree to 5=strongly agree); psychological wellbeing (Center for Epidemiologic Studies Depression Scale, 10-item version [CES-D-10]^20^, rated 1=rarely to 4=most of the time); health behaviors (sleep duration categorized as <7, 7–8, or >8 hours; exercise frequency categorized as rarely/never, a few times per month, 1–2 times per week, 3–5 times per week, or daily; and tobacco use type and frequency); stressors experienced in the past month (checklist); help-seeking preferences; and mental health service awareness.

Semi-structured interviews were conducted 26–31 October 2025, in private meeting rooms at the university campus. Two participants (P1, P2) were interviewed twice to achieve greater depth; one participant (P3) completed a single extended interview with bilingual interpretation assistance due to limited Chinese proficiency. Interviews lasted 20 to 40 minutes per session. Total transcribed text: approximately 49000 characters.

The interview guide addressed: background and pathway to China; cultural adaptation experiences; academic adjustment and stressors; e-cigarette use history and current practices; use contexts and environments (dormitory, campus, public spaces); perceptions of social norms regarding female smoking/vaping in Indonesia versus China; family awareness and reactions; peer relationships; and health awareness. The interviewer probed specifically on use contexts (e.g. situations that prompted use), affective states before and after use, and the relationship between experienced stressors and use patterns.

Interviews were conducted primarily in Mandarin Chinese with code-switching to Indonesian and English as needed. All interviews were audio-recorded and transcribed verbatim by the researcher. Transcripts were reviewed against audio recordings for accuracy.

### Data analysis

Analysis followed the IPA procedures outlined by Smith et al.^16^. The process began with reading and re-reading each transcript multiple times alongside questionnaire data to achieve immersion and develop an overall sense of each participant’s account. Initial noting involved making detailed exploratory comments throughout each transcript, including descriptive comments (summarizing content), linguistic comments (noting specific language use, metaphors, and tone), and conceptual comments (engaging with interpretation and meaning at a more abstract level). Emergent themes were then developed by analyzing exploratory comments to identify themes capturing the psychological essence of passages, expressed as concise phrases reflecting both participant content and researcher interpretation. These emergent themes were examined for patterns and connections, resulting in clustering into main themes and subthemes through processes of abstraction, subsumption, and polarization. Steps were repeated for each participant with deliberate bracketing of themes from previous cases to allow new themes to emerge. Finally, cross-case analysis examined themes from all three cases for convergence and divergence, resulting in a final thematic structure representing shared and distinctive aspects of participants’ experiences.

No qualitative software was used; analysis was conducted manually using printed transcripts and handwritten annotations. Trustworthiness was enhanced through maintaining a reflexive journal documenting analytical decisions, preserving idiographic detail through presentation of individual participant characteristics, providing verbatim quotations to ground interpretations in participant voices, integrating questionnaire data to contextualize interview findings, and transparent reporting following SRQR guidelines.

## RESULTS

### Participant profiles

All three participants were female Chinese Indonesian college students aged 18–21 years, with intermediate Chinese proficiency (Hanyu Shuiping Kaoshi [HSK] 3–4). Each participant’s profile is summarized briefly below to provide idiographic context for the thematic analysis that follows (Table 1).

**Table 1:** Participant characteristics of three female Chinese Indonesian e-cigarette users at a University in Fujian Province, IPA qualitative study, China, October 2025

*Characteristics*	*P1*	*P2*	*P3*
**Demographics**			
Age (years)	18	20	21
Year of study	2nd	3rd	2nd
Generation of Chinese descent	≥5th	3rd	3rd
Religion	Catholic	Buddhist	Christian
Home language	Indonesian	Hakka	English
Chinese proficiency	HSK 3–4	HSK 3–4	HSK 3–4
**E-cigarette use**			
Current status	Dual user^a^	Current daily user	Current daily user^b^
Change since arriving in China	No change	Increased	Increased
Primary use environment	Various	Outside during social outings	Outside, not in the dormitory
**Acculturation difficulties^c^**			
Overall cultural adaptation	4	1	3
Academic teaching methods	5	4	3
Dealing with academic pressure	5	2	3
Making friends with Chinese students	4	2	3
Stressors experienced (past month)	Language, academic, financial, loneliness, andcareer	Financial, homesickness, loneliness	Language, academic, and financial
**Psychological well-being (CES-D-10)^d^**			
Felt depressed	3	2	3
Felt lonely	3	3	3
Trouble concentrating	4	3	3
Felt hopeful about the future	2	1	3
**Social support**			
Friends to share joys/sorrows^e^	4	2	3
Overall satisfaction with support^e^	3	1	3
**Health behaviors**			
Sleep duration	<7 h	<7 h	<7 h
Exercise frequency	Rarely/never	A few times/month	1–2 times/week
**Help-seeking**			
Help-seeking preference	Friends, self-regulation, would not seek help	Would not seek help	Professional therapist
Mental health service awareness	Aware but never used	Aware but never used	Aware but never used

a Primarily uses conventional cigarettes, with occasional e-cigarette use.

b Dual use with conventional cigarettes.

c Rated 1 (no difficulty) to 5 (very difficult).

d Rated 1 (rarely) to 4 (most of the time).

e Rated 1 (strongly disagree/very dissatisfied) to 5 (strongly agree/very satisfied).

P: participant. HSK: Hanyu Shuiping Kaoshi (Chinese Proficiency Test). CES-D-10: Center for Epidemiologic Studies Depression Scale, 10-item version.

P1 (aged 18 years, second-year student, Catholic, ≥5th generation Chinese descent, Fujian ancestral province) reported the greatest adaptation difficulties across multiple domains. She rated overall cultural adaptation as quite difficult (4/5), with academic teaching methods and academic pressure both at maximum difficulty (5/5). P1 was a dual user who primarily smoked conventional cigarettes but continued to use e-cigarettes occasionally. She had initially used e-cigarettes in Indonesia before transitioning primarily to conventional cigarettes after arriving in China, citing taste preferences. Her e-cigarette use frequency remained unchanged since arriving in China. P1 reported the broadest range of stressors (language, academic, financial, loneliness, and career concerns), rarely exercised, and slept less than seven hours per night.

P2 (aged 20 years, third-year student, Buddhist, 3rd generation, Guangdong ancestral province) reported minimal overall adaptation difficulty (1/5) despite finding academic teaching methods quite difficult (4/5). P2 was a current daily e-cigarette user who reported increased use since arriving in China. She used e-cigarettes exclusively and did not smoke conventional cigarettes. Notably, P2 reported being very dissatisfied with social support overall (score 1/5) and feeling hopeful about the future only rarely (score 1 on the CES-D-10 item). She indicated she would not seek help for psychological distress.

P3 (aged 21 years, second-year student, Christian, 3rd generation, unknown ancestral province) reported moderate difficulty across acculturation domains (3/5). P3 was a current daily e-cigarette user who also used conventional cigarettes periodically (dual user). She reported increased e-cigarette use since arriving in China and sourced e-cigarettes from Indonesia. P3 was the only participant who indicated willingness to see a professional therapist for psychological distress.

All three participants reported sleeping less than seven hours per night and feeling lonely often (score 3 on the CES-D-10 loneliness item). All were aware of university mental health services but had never used them.

### Thematic structure

Analysis yielded four main themes with their corresponding subthemes, presented in Table 2.

**Table 2:** Thematic structure from IPA of e-cigarette use experiences among three female Chinese Indonesian college students, Fujian Province, China October 2025

*Themes*	*Subthemes*
**Social initiation and peer-embedded use**	Peer influence as an entry point Continuation within co-national networks Limited integration with Chinese peers
**Acculturation stressors without stress-coping use**	Academic pressure and language barriers Loneliness and limited social support Sleep insufficiency and health behavior patterns Explicit disconnection from stress-coping
**Navigating gendered social norms**	Indonesia as a restrictive environment China as relatively permissive Use environments: outside dormitories Family concealment
**Habituated practice and flavor preferences**	Habit framing Cross-border product sourcing Divergent behavioral trajectories

### Theme 1: Social initiation and peer-embedded use

*Peer influence as an entry point*

All three participants traced their e-cigarette initiation to peer influence during Indonesian high school:

*‘I was also influenced by friends’* (P1)

confirming this occurred in high school. P2 similarly explained:

*‘The first time was in Indonesia, high school … Because of friends, I guess.’* (P2)

P3 described mixed-gender peer influence:

*‘Maybe because of friends … Boys and girls, yeah, both boys and girls.’* (P3)

When asked about initial use contexts, P2 clarified that she used it primarily with friends rather than alone during that period:

*‘No [not alone] … just with them.’* (P2)

Notably, when asked about the gender composition of influential peers, P1 clarified that female classmates were the primary influence, while P3 described a mixed-gender peer context. This pattern of peer-mediated initiation occurred within Indonesia’s largely unregulated e-cigarette market.

*Continuation within co-national networks*

After arriving in China, e-cigarette use continued primarily within Indonesian peer networks rather than expanding to include Chinese friends. P2 explained:

*‘My friends around me, I often hang out with them, and they also vape, so I probably just … [continued].’* (P2)

When asked about the composition of her social circle, P2 confirmed:

*‘Still Indonesian friends mostly, but friends from other countries or Chinese friends, I also have some.’* (P2*)*

*Limited integration with Chinese peers*

Participants described limited social integration with Chinese students. P3 reported that her close friendships in China were with ‘Indonesian friends’, describing relationships with Chinese students as ‘not very close’. Questionnaire data corroborated this pattern: P1 rated ‘making friends with Chinese students’ as quite difficult (4/5), while P2 found it less challenging (2/5) but still maintained primarily co-national friendships for e-cigarette-related socializing.

### Theme 2: Acculturation stressors without stress-coping use

Questionnaire and interview data revealed substantial acculturation challenges across all three participants. A notable pattern, however, was that participants explicitly described their e-cigarette use as unrelated to the acculturation stressors they reported.

*Academic pressure and language barriers*

Academic adjustment emerged as a major challenge. P1 reported the highest difficulty levels, rating both ‘adapting to teaching methods’ and ‘dealing with academic pressure’ at the maximum (5/5). P1 elaborated on language-related academic difficulties:

*‘At first, my Chinese proficiency wasn’t very high, so during class I couldn’t understand.’* (P1)

P2 similarly described academic challenges despite reporting lower overall adaptation difficulty:

*‘Because I’m a foreign student, the teacher speaks quite fast in class, I might not understand some things, and sometimes their handwriting is messy.’* (P2)

All three participants reported language barriers among their stressors on the questionnaire, and two specifically identified academic pressure.

*Loneliness and limited social support*

All three participants reported feeling lonely often (score 3) on the CES-D-10 loneliness item. P2 additionally reported being very dissatisfied with social support overall (score 1/5) and disagreed that she had friends at school with whom to share joys and sorrows (score 2/5). Interview data illuminated the loneliness dimension. P1 commented with apparent resignation:

*‘Not many friends.’* (P1)

P3 described a temporal pattern of adaptation:

*‘At my first year [I was homesick], but now I’m fine … You get used to the life here.’* (P3)

*Sleep insufficiency and health behavior patterns*

All three participants reported sleeping less than seven hours per night. Exercise patterns varied substantially: P1 reported rarely or never exercising, P2 exercised a few times monthly, and P3 exercised one to two times weekly. When asked about stress management strategies, P1 described ‘sharing with friends’ and ‘going out … or sleeping’. None of the participants mentioned e-cigarette use as a coping or stress management strategy when asked about how they dealt with difficulties.

*Explicit disconnection from stress-coping*

Despite experiencing substantial stressors – academic pressure, language barriers, loneliness, sleep insufficiency, and (for P2) social support dissatisfaction – participants explicitly denied that e-cigarette use served stress-coping functions. When asked directly whether e-cigarettes helped with stress, P3 was unequivocal:

*‘No, no, no. Maybe I use them just because I’m used to it … I use them not to release pressure – it’s just a habit.’* (P3)

P1, despite reporting the most severe adaptation difficulties, similarly denied the stress-use connection. P2 framed her use as leisure rather than coping:

*‘For me, it’s nothing special – just playing around, that’s all.’* (P2)

Participants’ accounts consistently positioned e-cigarette use within recreational and habitual frames rather than coping frames, though this self-characterization should be interpreted alongside the possibility that habituated use may serve implicit regulatory functions not captured by explicit self-report.

### Theme 3: Navigating gendered social norms

*Indonesia as a restrictive environment*

Participants consistently described Indonesia as holding strong prohibitions against female tobacco use. P2 articulated this comprehensively:

*‘In Indonesia, any ethnicity, any religion, if a woman smokes, the way others look at you is not good.’* (P2)

When asked specifically about religious influences, P2 confirmed that Muslim women face particular constraints but extended the prohibition beyond religious boundaries:

*‘Any ethnicity, any religion, if women smoke, it’s not good.’* (P2)

P3 captured the gendered dimension:

*‘Parents don’t want their children to smoke. And I’m a girl.’* (P3)

*China is relatively permissive*

In contrast to Indonesia, participants perceived China as holding more tolerant attitudes toward female smoking. Participants’ perception of China as more permissive operated across several dimensions. Reduced public stigma:

*‘But in China, it feels like if a girl smokes, it feels very normal … But in Indonesia, Indonesia doesn’t like girls smoking’* (P2)

Diminished family surveillance due to geographical distance expanded social spaces for evening outings: P2 noted increased nighttime socializing compared to Indonesia; and the absence of religious prohibition frameworks that reinforced gender-specific tobacco norms in Indonesia. This perceived permissiveness did not appear to directly increase participants’ e-cigarette use, but it may have influenced where and with whom they felt comfortable using.

*Use environments: Outside dormitories*

E-cigarette use occurred primarily outside dormitory spaces, channeled into external social contexts. P2 explained her typical use context:

*‘Just going out, I often go to the night market and such.’* (P2)

P3 similarly indicated minimal dormitory use. P2 noted that coming to China had changed her nighttime social patterns:

*‘Maybe in Indonesia I didn’t often go out at night. Then after coming to China, I’m often still outside at night.’* (P2)

*Family concealment*

All three participants engaged in some form of concealment regarding their tobacco/e-cigarette use with families, enabled by geographical distance and digital communication patterns. P2 stated directly:

*‘My family doesn’t know.’* (P2)

*‘Chinese-Indonesian families are stricter about children smoking.’* (P2)

P3 acknowledged that her parents knew about her past smoking:

*‘They know, they know. But they think I already stopped smoking.’* (P3)

P1 presented a similar pattern of mutual face-saving. These concealment practices are enabled by geographical distance – participants communicate with families primarily through digital platforms (WhatsApp, WeChat) that allow selective disclosure.

### Theme 4: Habituated practice and flavor preferences *Habit framing*

Participants consistently framed e-cigarette use as a habituated practice rather than functional coping. P3 stated:

*‘I use them not to release pressure – it’s just a habit.’* (P3)

P2 similarly characterized use as leisure:

*‘For me, it’s nothing special – just playing around, that’s all.’* (P2)

This habit framing persisted even when participants acknowledged awareness of health risks. None expressed strong quit intentions.

*Cross-border product sourcing*

Participants expressed clear preferences for fruit-flavored e-cigarettes, which are available in Indonesia but have been restricted in China since October 2022 ^15^. P2 liked fruit flavors, and explained her sourcing:

*‘No, in China they don’t sell it, so we have to bring it from Indonesia.’* (P2)

P3 described similar practices:

*‘I bring them from my country … lipstick-style pods.’* (P3)

When asked whether she had tried Chinese e-cigarette products, P3 confirmed she had but rejected them:

*‘I tried, but I don’t like it. The taste is different.’* (P3)

Participants’ reported sourcing practices illustrate how mobile populations may navigate divergent regulatory environments.

*Divergent behavioral trajectories*

The three participants exhibited distinct behavioral trajectories after arriving in China. P2 reported increased e-cigarette use, attributing this to peer environment and lifestyle changes:

*‘Because my friends around me, I often hang out with them, and they also vape.’* (P2)

P3 similarly reported increased use alongside periodic conventional cigarette use, sourcing e-cigarettes from Indonesia and supplementing with local cigarettes. P1 presented a distinctive trajectory. She had primarily transitioned to conventional cigarettes after arriving in China, citing taste preferences:

*‘Because Chinese cigarettes, I don’t really like that taste.’* (P1)

She continued to use e-cigarettes occasionally. The inconvenience of sourcing Indonesian products may have contributed to her shift toward conventional cigarettes as her primary product. These divergent trajectories suggest that cross-cultural transition affects e-cigarette use differently depending on individual acculturation profiles, product access, and peer environments.

## DISCUSSION

This IPA study yielded four key findings regarding e-cigarette use among female Chinese Indonesian college students in China: 1) e-cigarette use was initiated through peer influence in Indonesia and maintained within co-national networks in China; 2) despite experiencing substantial acculturation stressors, participants did not frame e-cigarette use as stress coping; 3) participants perceived China as more permissive toward female vaping than Indonesia, managing family expectations through concealment; and 4) use was framed as habituated practice, with flavor preferences driving cross-border product sourcing. These findings are discussed below in relation to existing literature.

### Acculturation stressors without stress-coping use

A central finding was the explicit disconnection participants drew between their substantial adaptation challenges and e-cigarette use. Questionnaire data documented significant stressor exposure: all participants frequently experienced loneliness, all reported insufficient sleep, multiple stressors were identified (academic, financial, language-related), and P2 reported very low satisfaction with social support. These patterns align with the comprehensive review of Smith and Khawaja^3^ identifying academic, sociocultural, and psychological challenges as common among international students, and the documentation of Li and Sun^5^ of sleep difficulties during acculturation.

Yet when asked directly about the relationship between stress and e-cigarette use, participants consistently denied causal connections. This finding diverges from the Kim et al.^5^ review documenting associations between acculturative stress and smoking among Asian populations. Our participants, instead, framed use as a habit, leisure, and social practice – described as functionally disconnected from their acknowledged difficulties.

Several interpretations warrant consideration, drawing on Schwartz et al.^21^ reconceptualization of acculturation as a multidimensional process. First, participants may genuinely experience use as a non-functional habit, having been initiated through social influence before migration and maintained through behavioral automaticity. Second, the timing of initiation – prior to migration, during Indonesian high school – means that e-cigarette use predated acculturation stressors entirely. Use patterns established before migration may persist through habit mechanisms without becoming linked to post-migration stressors^21^. Third, cultural scripts may shape how use is understood and articulated; framing e-cigarette use as casual leisure rather than problematic coping may reflect impression management or genuinely different meaning systems regarding nicotine use.

However, this finding should be interpreted with caution. Self-report may not fully capture implicit or automatic coping processes (e.g. affect regulation, boredom management, negative reinforcement) that are common in habituated substance use. The distinction between ‘habit’ and ‘implicit coping’ is analytically challenging, and future research using ecological momentary assessment or more intensive probing techniques may be better positioned to distinguish these processes.

### Gendered social norms and the acculturation paradox

Participants perceived China as more tolerant of female smoking than Indonesia, consistent with the ‘acculturation paradox’ documented among Asian immigrant women in Western contexts^9,10^. Chen et al.^9^ and An et al.^10^ found that higher acculturation among Asian American women was associated with increased smoking, attributed to relaxation of traditional gender prohibitions. Our findings extend this literature to a non-Western receiving context.

However, the mechanism may differ from traditional acculturation processes. Our participants were not adopting Chinese norms per se – their e-cigarette use predated arrival in China. Rather, the perceived permissiveness may have enabled maintenance of existing behaviors with reduced psychological conflict and channeled use into more visible public contexts. The Salant and Lauderdale^8^ critical review of acculturation measurement in Asian immigrant health research is relevant here, as they argue that acculturation is often measured inadequately, conflating exposure to host culture with adoption of host behaviors. Our participants were exposed to Chinese environments but maintained Indonesian peer networks and behavioral patterns. This aligns with the Schwartz et al.^21^ argument that acculturation involves bidimensional processes wherein heritage and receiving culture orientations operate independently. Heritage students studying in ancestral homelands may exhibit a distinct adaptation pattern that warrants nuanced theoretical treatment.

### Peer networks and behavioral maintenance across borders

Consistent with the Firth et al.^6^ finding of increased e-cigarette use during study abroad, two of our three participants reported increased use after arriving in China. However, the mechanism differs from that proposed by Firth et al.^6^. Rather than adopting host country behaviors, our participants maintained use within co-national networks that reproduce home country behavioral norms. Kim et al.^5^ emphasized peer networks as critical influences on tobacco use among Asian populations; our findings suggest that these networks continue to operate transnationally among international students.

### Strengths and limitations

The study strengths include a focus on an unexplored population at the intersection of multiple understudied dimensions; integration of questionnaire and interview data providing both contextual case profiles and qualitative depth; employment of IPA methodology appropriate for small-sample experiential research^16,17^; and adherence to SRQR reporting guidelines^18^.

The study has limitations. As a qualitative, cross-sectional study, this research cannot establish causal relationships between acculturation factors and e-cigarette use patterns. The sample of three participants, while appropriate for IPA methodology^16,19^ limits the generalizability of the findings. The fact that only three of 45 surveyed students met all inclusion criteria and consented to participate raises the possibility of selection bias; those who agreed to discuss a stigmatized behavior may differ systematically from those who declined. Language barriers affected data collection, particularly with P3. Self-report data may be subject to social desirability bias regarding a stigmatized behavior, and the interview protocol may not fully capture implicit coping processes that participants did not articulate as such. The sample represents students at one university in Fujian – experiences may differ in other locations or among male Chinese Indonesian students.

### Future research

These findings point to several areas warranting further investigation. Larger, multi-site studies are needed to examine whether the patterns observed here – particularly the disconnection between acculturation stress and e-cigarette use among pre-migration users – extend to broader international student populations. Longitudinal designs that track e-cigarette use trajectories from pre-migration through post-migration adjustment would help disentangle temporal relationships between acculturative processes and use patterns. Research using ecological momentary assessment could better capture situational use contexts and implicit coping processes that cross-sectional interviews may not detect. Additionally, studies examining male international students and students from different sending countries would help determine the scope of the gendered dynamics observed here.

## CONCLUSIONS

E-cigarette use among female Chinese Indonesian college students in China was initiated through peer influence in Indonesia and maintained within co-national networks after migration. Despite experiencing significant acculturation stressors – academic pressure, language barriers, loneliness, and sleep insufficiency – participants explicitly described their e-cigarette use as unrelated to these stressors, instead framing use as a habituated practice. Participants perceived China as more permissive toward female vaping than Indonesia, and managed family expectations through concealment strategies enabled by transnational communication patterns. Flavor preferences drove cross-border product sourcing from Indonesia, illustrating how mobile populations navigate divergent regulatory environments. Future longitudinal and multi-site studies with larger samples are needed to confirm these preliminary findings and to examine whether the patterns observed here extend to broader international student populations.

## Supplementary Material



## Data Availability

The data supporting this research are available from the authors on reasonable request.
