# Supplementary material for: E-cigarette use among female Chinese Indonesian college students in China: A qualitative interpretive phenomenological analysis from an acculturation perspective
Source: Tob Induc Dis. 2026 May 14;24:10.18332/tid/219000. doi: 10.18332/tid/219000 (PMC13173517; doi:10.18332/tid/219000)
Supplement: Supplementary file 1 [file TID-24-59-s1.pdf]

Supplementary Material

SRQR (Standards for Reporting Qualitative Research) Checklist

Study Title: *E-cigarette Use among Female Chinese Indonesian College Students in China: A Qualitative Interpretive Phenomenological Analysis from an Acculturation Perspective*

This checklist has been completed according to the Standards for Reporting Qualitative Research (SRQR) guidelines.[18]

Title and Abstract

No.	Item	Reported
S1	*Title: Concise description identifying the study as qualitative or indicating the approach*	*Title explicitly identifies study as "Qualitative," specifies IPA methodology and acculturation perspective*
S2	*Abstract: Summary of key elements*	*Structured abstract with Introduction, Methods, Results, Conclusions (approximately 275 words); includes study location, inclusion criteria, and researcher characteristics*

Introduction

No.	Item	Reported
S3	*Problem formulation: Description and significance; review of relevant theory and empirical work*	*Introduction with literature review on international students,*[3],[5] *tobacco use,*[4],[6] *operationalized acculturation framework,*[7],[8],[20] *Indonesia–China regulatory context*[11–15]*; scope clarification between tobacco and e-cigarette use; sharpened research gap statement*
S4	*Purpose or research question*	*Four research questions in Study Purpose section; RQ2 reworded for IPA interpretive alignment ("how do participants make sense of")*

Methods

No.	Item	Reported
S5	*Qualitative approach and research paradigm*	*IPA with Berry's*[7] *acculturation framework operationalized as mechanism for heritage-linked international students*
S6	*Researcher characteristics and reflexivity*	*Research Team and Reflexivity section*
S7	*Context*	*Context section: Fujian Province university, October 2024*
S8	*Sampling strategy*	*Purposive sampling from N=45 survey; four explicit inclusion criteria with defined current e-cigarette use; information power justification*[19]
S9	*Ethical issues*	*Ethical Considerations section; institutional ethics approval explicitly reported (approving body, reference number, date); written informed consent; additional confidentiality protections for stigmatized behavior*
S10	*Data collection methods*	*Questionnaire (case-level contextualization) and semi-structured interviews (49,000 characters); interviewer probed use contexts, affective states, and stress–use relationship*
S11	*Data collection instruments and technologies*	*Interview guide domains specified with probing questions; questionnaire instruments described with response categories and scoring; CES-D-10 referenced*
S12	*Units of study*	*Three participants; brief idiographic case summaries; Table 1 with comprehensive characteristics*
S13	*Data processing*	*Manual analysis using printed transcripts*
S14	*Data analysis*	*IPA procedure per Smith et al.*[16] *described as continuous prose (reading/re-reading, initial noting, emergent themes, cross-case analysis)*
S15	*Techniques to enhance trustworthiness*	*Reflexive journal; verbatim quotations; questionnaire integration; SRQR adherence*

Results

No.	Item	Reported
S16	*Synthesis and interpretation*	*Four superordinate themes with subordinate themes (Table 2); idiographic case summaries; literature references reserved for Discussion*
S17	*Links to empirical data*	*Verbatim quotations throughout; questionnaire data in Table 1 and text*

Discussion

No.	Item	Reported
S18	*Integration with prior work, implications, transferability*	*Discussion opens with key findings; integrates with Kim et al.,[4] Firth et al.,[6] Schwartz et al.,[20] Chen et al.,[9] An et al.[10]; Directions for Future Research section; interpretive caveat on non-coping claim*
S19	*Limitations*	*Strengths and Limitations subsection; explicitly addresses causal inference, generalizability, selection bias, and implicit coping limitations*

Other

No.	Item	Reported
S20	*Conflicts of interest*	*Declarations section; ICMJE form completed*
S21	*Funding*	*Fuqing Social Science Federation (Grant No. FQSK2510)*

Reference

O'Brien BC, Harris IB, Beckman TJ, Reed DA, Cook DA. Standards for reporting qualitative research: a synthesis of recommendations. Acad Med. 2014;89(9):1245-1251. doi:10.1097/ACM.0000000000000388

© 2026 Chen Y. et al.
